# Toward the optimal strategy for sustained weight loss in overweight cancer survivors: a systematic review of the literature

**DOI:** 10.1007/s11764-016-0594-8

**Published:** 2017-01-18

**Authors:** Meeke Hoedjes, Maartje M. van Stralen, Sheena Tjon A Joe, Matti Rookus, Flora van Leeuwen, Susan Michie, Jacob C. Seidell, Ellen Kampman

**Affiliations:** 1 Department of Health Sciences and the EMGO+ Institute for Health and Care Research, VU University Amsterdam, De Boelelaan 1085, 1081 HV Amsterdam, The Netherlands; 2 Department of Dietetics, Netherlands Cancer Institute, Amsterdam, The Netherlands; 3 Department of Epidemiology, Netherlands Cancer Institute, Amsterdam, The Netherlands; 4 Department of Clinical, Educational and Health Psychology, UCL Centre for Behaviour Change, University College London, London, UK; 5 Division of Human Nutrition, Wageningen University, Wageningen, The Netherlands

**Keywords:** Cancer survivors, Weight loss maintenance, Behaviour change techniques, Lifestyle intervention components

## Abstract

**Purpose:**

To gain more insight into the optimal strategy to achieve weight loss and weight loss maintenance in overweight and obese cancer survivors after completion of initial treatment, this systematic review aimed to provide an overview of the literature on intervention effects on weight, to describe intervention components used in effective interventions, to identify and synthesize behaviour change techniques (BCTs) and to assess the frequency with which these BCTs were used in effective interventions.

**Methods:**

Six databases were searched for original research articles describing weight changes in adult overweight cancer survivors after participation in a lifestyle intervention initiated after completion of initial treatment. Two researchers independently screened the retrieved papers and extracted BCTs using the BCT Taxonomy version 1.

**Results:**

Thirty-two papers describing 27 interventions were included. Interventions that were evaluated with a robust study design (*n* = 8) generally showed <5% weight loss and did not evaluate effects at ≥12 months after intervention completion. Effective interventions promoted both diet and physical activity and used the BCTs ‘goal setting (behaviour)’, ‘action planning’, ‘social support (unspecified)’ and ‘instruction on how to perform the behaviour’.

**Conclusions:**

The results of this first review on intervention components of effective interventions could be used to inform intervention development and showed a need for future publications to report long-term effects, a detailed intervention description and an extensive process evaluation.

**Implications for cancer survivors:**

This study contributed to increasing knowledge on the optimal strategy to achieve weight loss, which is recommended for overweight cancer survivors to improve health outcomes.

## Introduction

A large proportion of cancer survivors (i.e. people who are living with a diagnosis of cancer, including those who have recovered from the disease [1]) are overweight or obese. Overweight and obesity have been related to an increased risk of cancer recurrence and decreased survival in cancer survivors [2–4]. In addition, compared with individuals without a history of cancer, cancer survivors have an increased risk for cancer [5], diabetes mellitus type II and cardiovascular disease [6, 7] and may experience a poorer health-related quality of life [8, 9].

Adherence to dietary, physical activity and body weight recommendations have been associated with a better health-related quality of life and overall well-being and a decreased risk of cardiovascular disease, diabetes mellitus type II and mortality in cancer survivors [10–14]. Although a reduction of body weight to a body mass index (BMI) in the normal range is advised for overweight and obese cancer survivors [15–17], such a weight-loss goal is unrealistic for most overweight and obese individuals. A more feasible weight-loss target, such as a decrease of 5 to 10% in body weight, has been shown to result in clinically relevant health benefits [18, 19].

Lifestyle changes needed for intentional weight loss are difficult to achieve and maintain, particularly for cancer survivors since they are coping with physical and emotional consequences of cancer and its treatment such as fatigue, neuropathy, anxiety and depression. Therefore, appropriate support is needed. A large body of evidence has shown that various lifestyle interventions are effective in reducing weight on the short-term in overweight individuals [20], including cancer survivors. However, intervention effects on weight loss are typically not maintained in the long-term [21]. Therefore, there is a need for evidence-based interventions that promote sustained health behaviour changes leading to long-term weight loss maintenance, which can be defined as ‘intentional weight loss of at least 10% of body weight and maintenance of this weight loss for at least 1 year’ [21]. Although the first long-term results of intervention studies among cancer survivors suggest that weight loss and improvements in diet and physical activity can be maintained for 1 year [22, 23], the optimal strategy for long-term weight loss maintenance remains unknown [24].

To gain more insight into the optimal strategy for weight loss and weight loss maintenance in overweight cancer survivors, knowledge on effective intervention components is needed. Behaviour change interventions are often complex and consist of many interacting components [25] (such as ‘who delivers the intervention’, ‘to whom’, ‘how often’, ‘for how long’, ‘in what format’, ‘in what context’ and ‘with what content’) [26], and they are often poorly described in the scientific literature [26, 27]. This hinders the accumulation of scientific evidence for their effectiveness and the identification of effective intervention components and underlying behaviour change mechanisms [26, 27]. To promote precise reporting of complex interventions, the content of an intervention can be described by its potentially active ingredients or behaviour change techniques (BCTs). BCTs can be defined as ‘observable, replicable and irreducible components of an intervention designed to alter or redirect causal processes that regulate behaviour’ [28]. The Behaviour Change Technique Taxonomy version 1 (BCTTv1) [28], a consensus-based, cross-domain hierarchically structured classificatory system, can be used as a reliable method to identify BCTs [29–31].

Although numerous reviews have been conducted on the effectiveness of lifestyle interventions in cancer survivors [32–40], little is known on the effectiveness of intervention components, including intervention content. Moreover, as all previous reviews focused on survivors of a single type of cancer, none of these reviews have focused on the effectiveness of lifestyle interventions for overweight survivors irrespective of cancer type, and none of these reviews have only included overweight cancer survivors and/or cancer survivors after completion of initial treatment. To gain more insight into the optimal strategy to achieve weight loss and weight loss maintenance in overweight and obese cancer survivors after completion of initial treatment, this systematic review aimed to provide an overview of the literature on intervention effects on weight, to describe intervention components used in effective interventions, to identify and synthesize BCTs and to assess the frequency with which these BCTs were used in effective interventions.

## Methods

### Literature search

A systematic review of the literature was conducted. Six databases (PubMed, Embase, Psychinfo, Web of Science, Cinahl and Central) were searched for relevant papers in January 2016. The following search terms were used: ((‘nutritional status’ OR (‘nutritional’ AND ‘status’) OR ‘nutrition’ OR ‘nutritional sciences’ OR (‘nutritional’ AND ‘sciences’) OR ‘diet’ OR ‘dietary’ OR ‘dietary supplements’)) AND (‘neoplasms’ OR ‘cancer’ OR ‘oncology’) AND (‘cancer patients’ OR ‘cancer survivors’) AND ((‘Intervention Studies’ OR ‘intervention’ OR ‘counselling’ OR ‘counseling’ OR ‘nutritional support’ OR (‘nutritional’ AND ‘support’) OR (‘nutrition’ AND ‘support’) OR ‘nutrition support’ OR ‘health promotion’)).

### Selection procedure

References that were retrieved from the database searches were exported to Endnote X5 and combined into one database with all retrieved references. Of duplicate references one was deleted. Two researchers (STAJ and MH) simultaneously and independently screened and labelled the titles, abstracts and the full-texts of all retrieved papers. First, the titles of the retrieved papers were screened and labelled in Endnote. If at least one of the researchers indicated that an abstract should have been read based on screening the title, both researchers subsequently read the abstract. Second, abstracts were screened and labelled in Endnote. If at least one of the researchers indicated that a full-text should have been read based on screening the abstract, both researchers subsequently read the full-text of the article. Finally, full-texts were read and labelled independently by both researchers.

We included original research articles describing the results of a lifestyle intervention (including a diet component) in adult (≥18 years) overweight (BMI ≥ 25) cancer survivors. Since it is expected that readiness to adopt long-term health behaviour changes is enhanced after completion of initial treatment when patients are primarily coping with the treatment and its side effects, only interventions that have been applied after completion of initial treatment (i.e. surgery, chemotherapy and radiotherapy) were included. Hormonal therapy was not considered to be initial treatment.

Because lifestyle interventions without a diet component are not likely to be able to achieve long-term weight loss maintenance, lifestyle interventions aiming to promote exercise or physical activity alone were excluded. Furthermore, a paper was excluded when it described non-human research, when a paper was not written in the English language, when it did not involve a lifestyle intervention, when the study population did not consist of overweight cancer survivors only, when the paper did not involve original research, when the intervention was not delivered after completion of initial treatment, when the lifestyle intervention did not include a diet component, when no results of the intervention were described, when weight was not included as an outcome and when the study population was younger than 18 years of age. A paper was also excluded when no abstract or full-text was available (e.g. in case of a congress abstract).

Inconsistencies between the researchers with regard to whether or not a paper should have been included in the review were discussed until consensus on inclusion or exclusion of the paper was achieved.

### Data extraction

The following data were extracted from the included articles: first author, year of publication, country, study design, type of cancer, sample size (total sample size, and if applicable sample size of the intervention and the control group), sex (percentage of female participants), mean age with standard deviation (SD), time after diagnosis or treatment, dropout rate, duration and type of intervention (physical activity plus diet vs. diet only), follow-up after the end of the intervention, mean baseline BMI and body weight in kilogrammes (kg) with SD, mean weight change in kilogramme with SD and percent weight change from baseline (Table 1). Table 1 provides an overview of the effect of the included lifestyle interventions on weight loss and weight loss maintenance. An intervention was considered to be effective in inducing weight loss when mean weight loss from preintervention to postintervention was significantly (*p* < 0.05) higher in the intervention group compared with mean weight loss in the control group in a randomized controlled trial (RCT). In case an RCT compared two or more interventions (e.g. two different diets) or in single arm pretest-posttest studies, an intervention was considered to be effective when a significant difference (*p* < 0.05) in weight between preintervention and postintervention was found. An intervention was considered to be effective in inducing long-term weight loss maintenance when mean weight loss from preintervention to 1 year postintervention was significantly (*p* < 0.05) higher in the intervention group compared with mean weight loss in the control group or when significant (*p* < 0.05) weight loss was found from baseline to 1 year follow-up after the end of the intervention for single arm studies. Long-term weight loss maintenance was defined as weight loss of at least 10% of body weight maintained for at least 1 year [21]. Study results were interpreted in the context of study design. An RCT with a usual care control group, an attention control or a less intensive intervention control group was considered to be the preferred study design with regard to interpretation of the effectiveness of the study and is referred to as a robust study design.

**Table 1:** Overview of study characteristics of the included studies (*n* = 32) and changes in weight during and after the included lifestyle interventions (*n* = 27)

First author (year) Country	Study design	Sample characteristics	Dropout rate	Intervention duration; type (name)	Follow up after the end of the intervention	Mean baseline BMI and weight in kilograms (SD)	Mean weight change in kilograms (SD) [%weight change from baseline]	Remarks
*Colorectal cancer survivors*								
Anderson (2010) [41] UK	Single arm pretest-posttest	*N* = 20 50% female Mean age: 61.1 (SD 9.0) 6–46 weeks postoperation	10%	3 months PA + diet (LiveWell)	No	BMI: 31.2 (5.4); no mean baseline body weight reported	−1.2 (4.4) (*p* value not mentioned)	Feasibility study; baseline weight not mentioned; weight is not a primary outcome. [% weight change cannot be calculated]
*Colorectal, breast and prostate cancer survivors*								
Morey (2009) [42] USA	RCT, wait-list control	*N* = 641 I: *N* = 319 DI: *N* = 322 I: 54.3% female DI: 56.3% female	I: 15.7% DI: 10.2%	12 months PA + diet; (RENEW)	No	BMI: I: 29.1 (SE 0.2) DI: 29.2 (SE 0.2) Weight: I: 85.7 (SE 0.7) DI: 84.7 (SE 0.7)	12 months vs. baseline: I: −2.06 (SE 0.19) [−2.40%] DI: −0.92 (SE 0.2) [−1.1%] I vs. DI***	I = intervention; DI = delayed intervention, initiated at 12 months after baseline Physical function as primary outcome
Demark-Wahnefried (2012) [43] USA		Mean age: I: 73.0 (SD 5.2) Mean age: DI: 72.9 (SD 5.0) Mean time since diagnosis: I: 8.7 (SD 2.8) years DI: 8.6 (SD 2.6) years	I: 24% DI: 24%		12 months		24 vs. 12 months: I: 0.25 (95% CI: −0.17; 0.67) NS [−2.61%] DI: −1.46 (95% CI: −1.97; −0.95)*** Weight 24 months vs. baseline: I: −2.24*** DI: −2.4***	I vs. DI not assessed at 24 months; weight change between baseline and 24 months calculated
*Postmenopausal breast cancer survivors*								
Befort (2012) [44] USA	Single arm pretest-posttest	*N* = 34 100% female Mean age: 58.9 (SD 7.8) Mean time since treatment: 3.1 (SD 1.6) years	9%	6 months PA + diet	No	BMI: 34.1 (4.4) Weight: 89.8 (13.6)	−12.5 (5.8)*** [−13.9%]	Feasibility study; dropout: attended ≤75% of intervention sessions and completed posttreatment data collection visits
Campbell (2012) [45] Canada	Single arm pretest-posttest	*N* = 14 100% female Mean age: 54.6 (SD 8.3) Mean time since treatment: 24.1 (SD 21.5) months	0%	24 weeks PA + diet	12 weeks	BMI: 30.1 (3.6) Weight: 78.8 (10.7)	24 weeks vs. baseline: −3.8 (5.0)** [−4.82%] 36 vs. 24 weeks: −0.8 (1.2)* [−1.0%] 36 vs. baseline −4.6 kg [−5.84%]	Feasibility study
Thompson (2015) [46] USA	3-Arm non-randomized controlled trial	*N* = 249 100% female Mean age: 54.9 (9.2) Time since treatment: ≥4 months LC: 81 LF: 93 C: 75	LC: 18.5% LF: 21.5% C: 29.3%	6 months Diet only	No	BMI: LC: 29.4 (2.5) LF: 28.2 (2.4) C: 29.2 (2.7) Weight: LC: 79.7 (8.6) LF: 77.6 (7.7) C: 79.7 (9.3)	6 months vs. baseline: LC: −10.5 (−11.6; −9.3) [−13.2%] LF: −9.3 (−10.3; −8.3) [−12.0%] C: −0.4 (−1.0; 0.3) [−0.5%] LF vs. control*** LC vs. control***	LF = low fat diet LC = low carbohydrate diet C = control Similar results described in Thompson et al. [47]
Thomson (2010) [48] USA	RCT	*N* = 43 LF: *N* = 22 LC: *N* = 21 100% female Mean age: 56.2 (SD 9.4) Mean time since diagnosis: 3.7 (3.4) years	7.5% LF: 4.5% LC: 9.5%	6 months Diet only	No	BMI: LC: 32.5 (4.7) LF: 31.0 (3.9) Weight: LC: 84.9 (14.0) LF: 83.1 (10.5)	24 weeks vs. baseline: LC: −5.9 (4.1)*** [−6.95%] LF: −6.3 (5.6)*** [−7.58%] LC vs. LF NS	LF = low fat diet LC = low carbohydrate diet: modified Atkins/reduced carbohydrate diet
De Waard (1993) [49] The Netherlands	RCT	Dutch sample: *N* = 54 (I: 30; C: 24) Polish sample: *N* = 48 (I: 29; C: 19) 100% female Age: 50–69 years Included directly after treatment	Dutch sample: 1 year I: 7%; C: 0% 3 years I: 40%; C: 37.5% Polish sample: 1 year I: 6.9%; C: 21.1%	12 months Diet only	Dutch sample: 2 years Polish sample: No	No mean baseline body weight and BMI reported	Dutch sample: (3 years vs. baseline) I: median −6 C: median +1 I vs. C*** Polish sample: (1 year vs. baseline) I: median −6 C: median −1 I vs. C***	Feasibility study; two samples: one Dutch and one Polish sample [% weight change cannot be calculated]
*Breast cancer survivors*								
Demark-Wahnefried (2014) [50] USA	RCT	*N* = 68 Individually tailored: 25 Team tailored: 25 Control: 18 100% female Mean age: 61.3 (7.4) Mean time since diagnosis: 24 (13) months	7.4% Individual: 8% Team: 8% Control: 5.6%	12 months PA + diet (DAMES)	No	BMI: Individual: 31.6 (3.4) Team: 30.8 (3.3) Control: 30.7 (2.6) Weight: Individual: 83.2 (8.8) Team: 82.6 (13.4) Control: 81.6 (9.3)	12 months vs. baseline: Individual: −3.77 (4.80) [−4.5%] Team: −2.09 (4.30) [−2.5%] Control: −0.87 (2.97) [−1.1%] Team vs. control: NS Individual vs. control*	Feasibility study; groups consist of mother-daughter dyads; each comprised a survivor of breast cancer and her adult biological daughter. Only results for cancer survivors are reported here.
Djuric (2009) [51] USA	RCT	*N* = 31 enrolled; *N* = 24 randomized after 6 months 100% female Dietitian group: *N* = 12 Mean age: 56 (SD 10) Mean time since diagnosis: 5.6 (SD 4.3) years Spirituality group: *N* = 12 Mean age: 55 (SD 8) Mean time since diagnosis: 5.7 (SD 3.3) years	8.4% at 18 months Dietitian group: 8.4% Spirituality group: 8.4%	18 months PA + diet vs. PA + diet + spirituality counselling	No	BMI: Dietitian group: 36 (5) Spirituality group: 36 (3) Weight: Dietitian group: 94.9 (14.8) Spirituality group: 93.8 (11.3)	Dietitian group: 0–6 months: −2.6 (4.7) 6–18 months: +0.4 (3.0) 0–18 months: −2.2 [−2.32%] Spirituality group:0–6 months: −1.0 (7.0) 6–18 months: +0.3 (3.4) 0–18 months: −0.7 [−0.75%] Dietician only vs. dietician and spirituality 18 vs. 6 months: NS	Pilot-study; randomization after 6 months of PA + diet counselling (dietitian-led counselling); dietitian-led counselling vs. dietitian-led counselling + spirituality counselling *p* Values for within-group changes are not reported.
Flynn (2010) [52] USA	RCT	*N* = 44 100% female Mean age: 59.2 (SD 6.1) Included within 4 years after completing treatment	36.4% at 16 weeks; 54.5% at 16 weeks +6 months	2 × 8 weeks Diet only: randomized diet + 6 months diet of choice	No	BMI: 27.9 (2.8); no mean baseline body weight reported	16 weeks vs. baseline: NCI: −2.7 (1.4) [−3.9%] PBOO: −3.6 (1.9) [−4.9%] NCI vs. PBOO* Weight at 16 weeks + 6 m: 66.9 (8.7) Weight at 16 weeks: 68.0 (8.8) 16 weeks + 6 m vs. 16 weeks: *p* = 0.07% weight change was greater for NCI when NCI was consumed first and greater for PBOO when PBOO was consumed first, both***.	2 × 8-week diet; random assignment of diet order + 6 months diet of choice NCI = National Cancer Institute Diet PBOO = Plant-based olive oil diet *p* Values for within-group changes are not reported.
Greenlee (2013) [53] USA	RCT, wait-list control	*N* = 42 100% female IA: *N* = 22 Mean age: 52.6 (SD 8.0) Mean time since diagnosis: 3.5 (SD 2.1) years WCA: *N* = 20 Mean age: 48.6 (SD 9.6) Mean time since diagnosis: 4.7 (SD 3.2) years	IA: 4.5% WCA: 15%	6 months PA + diet (Curves program)	6 months	BMI: IA: 33.4 (6.6) WCA: 32.9 (5.2) Weight: IA: 85.1 (12.5) WCA: 83.8 (15.3)	6 months vs. baseline: IA: −2.87 (3.15)*** [−3.37%] WCA: −1.42 (2.50)* [−1.69%] IA vs. WCA* 12 months vs. baseline: IA: −1.76 (3.21)* [−2.07%] WCA: −2.14 (3.77)*	IA = immediate arm: 6 month- intervention followed by 6 months of observation; WCA = wait-list control arm: 6 months of observation followed by 6 months of intervention.
Harrigan (2015) [54] USA	RCT	*N* = 100 In-person: 33 Telephone: 34 Usual care: 33 100% female Mean age: 59.0 (7.5) Mean time since diagnosis: 2.9 (2.1) years	6 months: In-person: 9.1% Telephone: 29.4% Usual care: 6% 12 months: In-person: 33.3% Telephone: 55.9% Usual care: 42%	6 months PA+ diet (LEAN)	6 months*	BMI: In-person: 33.5 (6.7) Telephone: 31.8 (5.4) Usual care: 34.0 (7.5) Weight: In-person: 88.1 (18.3) Telephone: 84.3 (15.3) Usual care: 90.4 (20.3)	6 months vs. baseline: In-person: −5.6 (−7.1; −4.1) [−6.4%] Telephone: −4.8 (−6.5; −3.1) [−5.7%] Usual care: −1.7 (−3.2; −0.3) [−1.9%] In-person vs. usual care:** Telephone vs. usual care:** In-person vs. telephone NS 12 months vs. baseline (self-reported) In-person: −5.6 (−8.0; −3.3) [−6.3%] Telephone: −6.3 (−9.9; −2.6) [−7.7%] Usual care: −3.8 (−5.6; −1.9) [−4.3%] In-person vs. usual care: NS Telephone vs. usual care: NS In-person vs. telephone: NS	In-person vs. telephone weight loss counselling vs. usual care. *6-month follow-up measurement of self-reported weight only*; weight was measured at baseline and directly after the end of the intervention.
Jen (2004) [55] USA	RCT	*N* = 48 C: *N* = 13 WW: *N* = 11 Ind: *N* = 13 Comp: *N* = 11 100% female Mean age: 51.7 (SD 8.4) Time since diagnosis: up to 4 years	18.8% C = 7.7% WW = 27.3% Ind = 30.8% Comp = 9.1%	12 months Diet only vs. diet and PA	No	BMI: C: 34.9 (SE 1.2) WW: 35 (SE1.2 ) Ind: 35.5 (SE 1.1) Comp: 36.8 (SE 1) Weight: C: 95.0 (SE 3.6) WW: 95.5 (SE 5) Ind: 91.4 (SE 2.7) Comp: 100.5 (SE 5)	12 months vs. baseline: C: +1.1 (SE 1.7) [+1.2%] WW: −2.7 (SE 2.1) [−2.83%] Ind: −8.0 (SE 1.9)* [−8.75%] Comp: −9.5 (SE 2.7)* [−9.45%] Between-group effect:***	Pilot study; C = control WW = weight watchers Ind = individualized Comp = comprehensive; Comprehensive group = both individualized counselling and weight watchers. Baseline values between groups NS. Similar results presented in Djuric et al. [56].
McTiernan (1998) [57] USA	Single arm pretest-posttest	*N* = 10 100% female Age: 40–74 years 1–5 years posttreatment	10%	8 weeks PA + diet	No	BMI not mentioned 76.7 (SD not mentioned)	−1.18 (1.4)* [−1.54%]	Pilot study
Mefferd (2007) [58] USA	RCT	*N* = 85 100% female Mean time since diagnosis: 3.5 (SD 3.0) years I: *N* = 56 Mean age: 56 (SD 9) C: *N* = 29 Mean age: 56 (SD 8)	10.6% 16.1% 0%	16 weeks PA + diet	No	BMI: I: 30.7 (3.8) C: 31.3 (4.8) Weight: I: 83.9 (11.9) C: 86.3 (14.2)	16 weeks vs. baseline: I: −5.7 [−6.79%] C: −0.5 [−0.6%] I vs. C*	Weight change calculated; no SD mentioned. This study has a wait-list control design. However, only results directly after the end of the intervention are described. Similar results are described in Pakiz et al. [59].
Patella (2009) [60] Italy	Single arm pretest-posttest	*N* = 97 100% female Mean age: 57 (SD 9.9) Mean time since surgery in those diagnosed ≤5 years ago: 17.6 (SD 15.8) months; *n* = 76 Mean time since surgery in those diagnosed >5 years ago: 137.2 (SD 78.8) months; *n* = 16	22.8%	12 months Diet only	No	BMI: 30.6 (4.2) Weight: 78.5 (9.7)	−6.6 (3.7)*** [−8.41%]	Study was originally designed as a two arm pretest-posttest study; due to much larger dropout rate in the control group (73.2%) compared with the Intervention group (22.8%), only pretest-posttest results of the intervention group are presented.
Rock (2015) [61] USA	RCT	*N* = 697 100% female I: 348 Mean age: 56 (9) Mean time since treatment: 2.02 (0.55) years C: 349 Mean age: 56 (9) Mean time since treatment: 2.18 (0.55) years	I: 13.8% C: 17.8%	24 months PA + diet (ENERGY)	No	BMI: I: 31.6 (4.7) C: 31.4 (4.6) Weight: I: 85.0 (14.3) C: 84.7 (13.8)	12 months vs. baseline: I: −5.3 [−6.0%] C: *−*1.2 [−1.5%] I vs. C*** 24 months vs. baseline: I: −3.6 [−3.7%] C: *−*0.9 [−1.3%] I vs. C***	
Saquib (2009) [62] USA	RCT	*N* = 1760* 100% female C: *N* = 760* Mean age: 54.5 (SD 8.4) Mean time since diagnosis: 25.3 (SD 12.2) months I: *N* = 750* Mean age: 54.4 (SD 8.4) Mean time since diagnosis: 24.5 (SD 12.2) months	14%	4 years Diet only Women’s Healthy Eating and Living (WHEL) Study	No	BMI: I: 30.7 (4.8) C: 31.0 (5.5) Weight: I: <55 years: 83.7 ≥55 years: 81.0 C: <55 years: 83.2 ≥55 years: 82.7	I: <55: year 1: −0.1; year 2 or 3: +1.5; year 4: +1.9 year 4 vs. baseline: [+2.27%] ≥55: year: −0.7; year 2 or 3: +0.2; year 4: +0.5 year 4 vs. baseline: [+0.62%] C: <55: year 1: +0.8; year 2 or 3: +1.6; year 4: +1.6 year 4 vs. baseline: [+1.92%] ≥55: year 1: −0.3; year 2 or 3: −0.4; year 4: −0.6 year 4 vs. baseline: [−0.73%] NS difference in mean body weight between the groups either at baseline or at follow-up. Weight change difference I vs. C*** in year 1 only	*Subgroup analyses of 1510 participants in the WHEL Study: overweight and obese participants (BMI ≥ 25) only; results for weight are stratified for age. Weight is not a primary outcome. No statistically significant difference in weight between intervention and control directly after intervention completion.
Sheppard (2016) [63] USA	RCT	*N* = 31 I: 15 C: 16 100% female Mean age: 54.7 (9.8) Mean time since treatment: 1.7 (0.88) years	I: 25% C: 33.3%	12 weeks PA + diet (Stepping STONE)	No	BMI: I: 35.2 (4.8) C: 37.4 (8.6) Weight: I: 98.2 (19.5) C: 97.8 (21.1)	12 weeks vs. baseline: I: −0.77 [−0.78%] C: + 0.18 [+0.18%] *p* Value I vs. C not mentioned	Feasibility study; this study has a wait-list control design. However, only results directly after the end of the intervention are described.
Spark (2015) [64] Australia	Single arm pretest-posttest	*N* = 29 100% female Mean age: 54.9 (8.8) Mean time since treatment: 7.1 (1.4) months	21%	6-month intervention + 6 month-extended intervention PA +diet	6 months	BMI: 30.0 (4.2) Weight: 81.8 (13.1)	6 months vs. baseline: −5.5* [−6.7%] 12 months vs. baseline: −4.2* [−5.1%] 18 months vs. baseline: −4.2 (−6.0; −2.4)* [−5.1%] 12 vs. 6 months: + 1.3 (−0.5; 3.1) NS [+1.6%] 18 vs. 12 months: −0.1 (−1.9; 1.8) NS [−0.1%]	Feasibility study; 6-month extended contact intervention after the original 6-month PA + diet intervention.
Stolley (2009) [65] USA	Single arm pretest-posttest	*N* = 23 100% female Mean age: 51.4 (SD 8.9) Time since treatment: ≥6 months	13%	6 months PA +diet (Moving Forward)	No	BMI: 34.1 (95% CI 30.8; 37.4) Weight: 87.8 (95% CI 79.2;96.3)	−2.53 (−3.91; −1.14)*** [−2.88%]	
Swisher (2015) [66] USA	RCT	*N* = 28 100% female Mean time since diagnosis: 4–5 years I: 18 Mean age: 53.8 C: 10 Mean age: 53.6	I: 27.8% C: 0%	12 weeks PA + diet (Get Fit for the Fight)	No	BMI: I: 30.9 (3.3) C: 32.5 (7.1) Weight: I: 80.2 (9.6) C: 85.4 (21.4)	12 weeks vs. baseline: I: −3.0 [−3.7%]* C: −0.4 [−0.5%]	I vs. C: % body fat*
Travier (2014) [67] Spain	Single arm pretest-posttest	*N* = 42 100% female Mean age: 54.8 (SD 8.7) Mean time since treatment: 87.6 (SD 62.9) days	12%	12 weeks PA + diet	No	BMI: 30.5 (3.9) Weight: 73.3 (10.2)	−7.8 (2.9)*** [−10.6%]	Feasibility study; similar results are presented in Travier et al. [68]
Vitolins (2014) [69] USA	Single arm pretest-posttest	*N* = 19 100% female Median age (range): 59 (38–72) Time since treatment: ≥6 months	10.5%	12 weeks PA + diet	No	BMI: Median 31.3 Weight: 88.0 (18.3)	−6.3 (3.6)*** [−7.16%]	Feasibility study
*Breast and endometrial cancer survivors*								
McCarroll (2015) [70] USA	Single arm pretest-posttest	*N* = 50 100% female Mean age: 58.4 (10.3) Time since treatment: ≥ 6 months	15%	1 month (Lose It!) PA + diet	No	BMI: 36.4 (8.1) Weight: 97.3 (22.5)	−2.3*** [−2.4%]	Feasibility study
*Endometrial cancer survivors*								
Von Gruenigen (2008) [71] USA	RCT	*N* = 45 100% female I: *N* = 23 Mean age: 54 (2.0) Time since diagnosis: median 20.6 months C: *N* = 22 Mean age: 55.5 (1.6) Time since diagnosis: median 26.7 months	I: 22% C: 10%	6 months PA + diet	6 months	BMI: I: 43.5 (SE 2.1) C: 41.1 (SE 2.2) Weight: I: 115.4 (29.4)C: 107.1 (24.7)	I: 3 months vs. baseline: −2.6 (95% CI: −1.0 to −4.2), *p* = 0.001 6 vs. 3 months: −0.3 (95% CI: −1.1 to 1.8) NS 12 vs. 6 months: −0.3 (95% CI: −2.8 to 3.3) NS 6 months vs. baseline: −2.9 kg [−2.51%] C: no significant weight changes from baseline 12 months vs. baseline: I: −3.5 [−3.03%] C: +1.4[+1.3%] I vs. C: −4.9 (95% CI −9.0, −0.9)*	
Von Gruenigen (2012) [72] USA	RCT	*N* = 75 100% female I: *N* = 41 Mean age: 57.0 (SD 8.6) Time since diagnosis: median 17.6 months (range: 5.5–36.0) C: *N* = 34 Mean age: 58.9 (SD 10.9) Time since diagnosis: median 25.5 months (range 4.4–36.0)	I: 14.6% C: 29.4%	6 months PA + diet (SUCCEED)	6 months	BMI: I: 36.4 (5.5) C: 36.5 (9.6) Weight: I: 95.7 (19.0) C: 94.0 (23.0)	6 months vs. baseline: I: −3.9 [−4.08%] C: +0.6 [+0.64%] I vs. C*** 12 months vs. baseline: I: −3.0 [−3.13%] C: +1.4 [+1.49%] I vs. C***	

Interventions that were tested using a randomized controlled trial with a usual care control group, not in the context of a feasibility or pilot study are marked with grey colouring

*NS* not significant, *I* intervention group, *C* control group, *PA* physical activity, *BMI* body mass index, *SD* standard deviation, *SE* standard error, *95% CI* 95% confidence interval, *RCT* randomized controlled trial

*p < 0.05; **p < 0.01; ***p < 0.001

The following characteristics of the included lifestyle interventions were extracted and described in Table 2: the aims of the intervention, the theoretical framework on which the intervention was based, a description of the control condition and details on intervention components [26], such as by whom the intervention was delivered, the frequency and length of intervention contacts, the format of intervention contacts, the context in which the intervention was delivered and the content of the intervention. BCTs were used to describe the content of the intervention.

**Table 2:** Description of intervention characteristics of included interventions that have been shown to be effective after evaluation in a robust study (*n* = 8)

First author (year), country	Intervention aims and components^a^	Control condition	Theoretical framework	Behaviour change techniques^b^
*Colorectal, breast and prostate cancer survivors*				
Morey (2009) [42] UK	*Aims*: weight loss goal of 10% during the 12-month study period; restriction of saturated fat to less than 10% of energy intake; consumption of at least seven servings (for women) or nine servings (for men) of fruits and vegetables per day; 15 min of strength training exercise every other day and 30 min of endurance exercise each day. *Who delivers the intervention*: health counsellor *How often*: quarterly newsletters, 15 telephone counselling sessions (15 to 30 min) and 8 prompts: weekly during the first 3 weeks, every other week for 1 month and then monthly. *For how long*: 12 months *In what format*: mailed print materials (personally tailored workbook and tailored two-page progress report newsletters) and a program of individual telephone counselling and automated telephone prompts. Personalized workbook with bar graphs comparing participants’ current lifestyle behaviours and weight status with recommended levels. Workbook chapters provided standardized content on exercise and a healthy calorie-restricted diet. Participants received a pedometer, exercise bands, an exercise poster depicting six lower extremity strength exercises, a table guide to food portioning and personalized record logs to self-monitor daily exercise and dietary intake. *In what context*: home-based	Delayed intervention, wait-list control.	Social cognitive theory [73] Transtheoretical model [74]	-Goal setting (behaviour) -Problem solving -Goal setting (outcome) -Action planning -Review outcome goal(s) -Feedback on behaviour -Self-monitoring of behaviour -Social support (unspecified) -Instruction on how to perform the behaviour -Demonstration of the behaviour -Prompts/cues -Credible source -Social reward -Adding objects to the environment
*Breast cancer survivors*				
Greenlee (2013) [53] USA	*Aims*: *Diet*: reduce caloric intake (1200 cal/day for 1 to 2 weeks, followed by 1600 cal/day) and to distribute calorie intake as 45% protein/30% carbohydrates/25% fat. *Exercise*: 3 days/week, 30-min sessions while maintaining 70–75% of maximal heart rate. *Who delivers the intervention*: an instructor (diet) and a trainer (exercise), both curves staff (commercial Curves Weight Management Program) *How often*: nutrition course consisted of six 1-h weekly group sessions; weekly motivational telephone calls; three to five 30-min personally tailored exercise sessions per week. *For how long*: 6 months *In what format*: group sessions plus individual telephone counselling. Participants were provided with a Curves weight loss program instruction and recipe book, DVDs and an instructor’s manual. Participant b was also provided with Polar S-610 heart rate monitors (Polar Electro Oy, Finland) to monitor and record heart rate. Dietary sessions started ~1 month after the exercise program. *In what context*: Columbia University Medical Center (nutrition course), Curves fitness centre (exercise sessions).	In the wait-list control arm, participants were observed for 6 months during which they were asked not to change their physical activity or diet, followed by 6 months of the Curves program. In the immediate arm, participants received 6 months of the Curves weight loss program, followed by 6 months of observation during which they could engage in any diet and physical activity of their choice.	Not mentioned	-Goal setting (behaviour) -Goal setting (outcome) -Action planning -Biofeedback -Social support (unspecified) -Instruction on how to perform the behaviour -Demonstration of the behaviour -Behavioural practice/rehearsal -Graded tasks -Adding objects to the environment
Harrigan (2015) [54] USA	*Aim*: *Diet*: reduce energy intake to the range of 1200 to 2000 kcal/day based upon baseline weight and to incur an energy deficit of 500 kcal/day. The dietary fat goal: 25% of total energy intake. *Physical activity*: 150 min per week of moderate-intensity activity; 10,000 steps per day. *Who delivers the intervention*: a registered dietician (Certified Specialist in Oncology Nutrition and trained in exercise physiology and behaviour modification counselling) *How often*: 11 30-min individualized counselling sessions once per week in month 1, every 2 weeks in months 2 and 3, and once per month in months 4, 5 and 6. *For how long*: 6 months *In what format*: Both the in-person and telephone groups received the same lifestyle intervention. Women were provided with a scale, a pedometer, a LEAN Journal, and an	The usual care group was provided with American Institute for Cancer Research nutrition and physical activity brochures and was also referred to the Yale Cancer Center Survivorship Clinic, which offers a two session weight management	Social cognitive theory [73] The weight loss intervention was adapted from the Diabetes Prevention Program, updated with 2010 US Dietary Guidelines, and adapted to the breast cancer survivor population using the American	-Goal setting (behaviour) -Action planning -Self-monitoring of behaviour -Self-monitoring of outcome(s) of behaviour -Social support (unspecified) -Instruction on how to perform the behaviour -Credible source -Adding objects to the environment *Usual care group*: –
Harrigan (2015) USA [54] Continued	11-chapter LEAN book to guide each session. *In-person group*: individual face-to-face counselling sessions; *Telephone group*: individual telephone counselling sessions. *In what context*: *In-person group*: home-based physical activity program; location dietary counselling not mentioned; *Telephone group*: home-based.	program. At the completion of the study, usual care participants were offered the LEAN book and LEAN Journal, as well as an in-person counselling session.	Institute for Cancer Research/World Cancer Research Fund and American Cancer Society nutrition and physical activity guidelines.	
Mefferd (2007) USA [58] and Pakiz (2011) USA [59]	*Aims*: *Primary goal*: facilitate a modest weight loss that is sustained, with an emphasis on features that increase this likelihood, such as acceptance of modest weight loss and focusing on skills for weight maintenance. *Physical activity*: muscle strengthening exercises 2–3 times per week and regular planned aerobic exercise, with an initial goal of daily activity and a step-wise increase in time and intensity with the overall long-term goal of ~1 h per day of moderate to vigorous physical activity. *Diet*: 500–1000 kcal/day deficit via reduced energy density of the diet plus avoidance of overly strict dieting behaviour that did not promote satiety or long-term maintenance. Participants were encouraged to include high-fibre vegetables, whole grains, fruit and adequate protein to meet nutritional needs and to contribute to satiety. *Who delivers the intervention*: Trained investigators and research staff *How often*: Closed group sessions: weekly for 4 months, and monthly follow-up sessions through 12 months. Individualized telephone-based counselling: weekly calls in the first month and every other week for the next 2 months and once a month thereafter. *It should be noted that both studies only report data collected at baseline and at 16 weeks.* *For how long*: 16 weeks *In what format*: Closed group sessions (with an average of 12–15 women per group) + individualized telephone-based counselling**.** A pedometer was provided. *In what context*: not mentioned	(Wait-list) control group was provided only general contact (monthly check-up calls, holiday and seasonal cards and mailed communications) without specific reference to weight management topics through a 12-month period of data collection. Following that period, they were provided all written intervention materials and a concise version of the didactic material, and facilitated discussion was offered in the format of a 2-day seminar.	Intervention curriculum was based on the new elements of cognitive behavioural therapy [75] for obesity in addition to many elements of standard behavioural treatment for obesity.	-Goal setting (behaviour) -Problem solving -Goal setting (outcome) -Action planning -Review behaviour goal(s) -Feedback on behaviour -Self-monitoring of behaviour -Social support (unspecified) -Instruction on how to perform the behaviour -Information about health consequences -Monitoring of emotional consequences -Demonstration of the behaviour -Behavioural practice/rehearsal -Graded tasks -Reduce negative emotions -Adding objects to the environment -Framing/reframing -Self-talk
Rock (2015) [61] USA	*Aims*: weight loss of at least 7% body weight (at 2 years). *Diet*: a deficit in energy intake of 500–1000 kcal/day relative to expenditure to promote a weight loss of 1–2 lb/week. *Physical activity*: The long-term goal was an average of at least 60 min/day of purposeful exercise at a moderate level of intensity. *Who delivers the intervention*: counsellors with backgrounds in dietetics, psychology and/or exercise physiology. *How often*: 4 months of weekly 1 h group sessions for closed-groups of an average of 15 women, tapering to every other week for 2 months. From 6 months onward, the groups met monthly for the remainder of the year; brief (10- to 15-min) personalized guidance delivered by telephone and/or e-mail: a total of approximately 14–16 counselling calls or contacts in the first study year and a total of 24–38 calls or messages during the two-year period of the intervention. Quarterly tailored print newsletters from 6 to 24 months. *For how long*: 24 months *In what format*: face-to-face closed-groups counselling sessions with individual telephone counselling, e-mail contact and individually tailored print newsletters. Materials and other items were provided: a participant notebook with worksheets, handouts and illustrations, food and exercise journals, a pedometer, books with caloric content of food, recommended web-based resources for monitoring intake and expenditure, a digital scale and two digital video discs for walking three and five miles. *In what context*: partly home-based; location of group sessions not mentioned.	Participants in the less intensive intervention control group were provided weight management resources and materials in the public domain. An individualized diet counselling session was provided at baseline and 6 months, and current physical activity recommendations (at least 30 min per day) were advised. They received monthly telephone calls and/or e-mails from the study coordinator and were invited to attend optional informational seminars on aspects of healthy living other than weight control every other month during the first year.	Behavioural determinants model [76], which is based on social cognitive theory [73]; motivational interviewing [77]; cognitive behavioural therapy [78]	-Goal setting (behaviour) -Problem solving -Goal setting (outcome) -Action planning -Review behaviour goal(s) -Feedback on behaviour -Self-monitoring of behaviour -Self-monitoring of outcome(s) of behaviour -Social support (unspecified) -Instruction on how to perform the behaviour -Demonstration of the behaviour -Behavioural practice/rehearsal -Graded tasks -Credible source -Non-specific reward -Avoidance/reducing exposure to cues for the behaviour -Adding objects to the environment -Framing/reframing *Control group:* -Goal setting (behaviour) -Action planning -Social support (unspecified)
Swisher (2015) [66] USA	*Aims*: *Physical activity*: 150 min per week of moderate-intensity aerobic exercise, defined as rating of perceived exertion of 11–14 (corresponding to 60–75% of peak heart rate achieved on the exercise test). *Diet*: decrease dietary fat caloric intake by 200 kcal per week. *Who delivers the intervention*: exercise physiologists trained in medical rehabilitation (for the supervised exercise sessions) and a dietician, a specialist in nutrition for cancer patients. *How often*: individually supervised, moderate-intensity 30-min aerobic exercise sessions three times per week and two unsupervised sessions per week at home; two individual dietary counselling sessions (at the start and approximately 1 month after initial counselling sessions). *For how long*: 12 weeks *In what format*: individually supervised aerobic exercise sessions and individual face-to-face dietary counselling. Exercise and food logs were provided. *In what context*: at an exercise facility (supervised exercise sessions); at home (unsupervised exercise sessions); location of dietary counselling not mentioned.	The control group received written materials about healthy eating for cancer survivors and suggestions on ways to achieve regular physical activity. They were not instructed to avoid diet change or exercise. However, they did not receive any specific counselling or supervision.	Not mentioned	-Goal setting (behaviour) -Action planning -Review behaviour goals -Discrepancy between current behaviour and goal -Monitoring of behaviour by others without feedback -Self-monitoring of behaviour -Social support (unspecified) -Instruction on how to perform the behaviour -Demonstration of the behaviour -Behavioural practice/rehearsal -Credible source *Control:* -Instruction on how to perform the behaviour
*Endometrial cancer survivors*				
Von Gruenigen (2008) [71] USA	*Aims*: 5% weight loss in 6 months. *Who delivers the intervention*: Registered dietician and the primary investigator *How often*: Weekly group contacts for 6 weeks, bi-weekly for 1 month and monthly for 3 months. Participants were contacted by phone or newsletter every week that the group did not meet. Individual face-to face contacts at 3, 6 and 12 months. *For how long*: 6 months *In what format*: Group + individual sessions face-to-face + contacted by phone or newsletter every week that the group did not meet. Pedometers were provided for patient feedback. Participants saw the primary investigator at 3, 6 and 12 months and received counselling regarding overall health concerns and reinforcement of specific group session topics. *In what context*: not mentioned	The usual care group received an informational brochure. To reduce attrition, they were offered a modest monetary incentive ($20.00) for each completed data collection point. The primary investigator saw the usual care group at 3, 6 and 12 months and provided counselling regarding overall health concerns. They did not receive any advice related to weight loss, physical activity or nutrition.	Social cognitive theory [73]	-Goal setting (behaviour) -Problem solving -Goal setting (outcome) -Action planning -Discrepancy between current behaviour and goal -Feedback on behaviour -Self-monitoring of behaviour -Social support (unspecified) -Instruction on how to perform the behaviour -Graded tasks -Non-specific reward -Reduce negative emotions -Adding objects to the environment *Control group:* -Instruction on how to perform the behaviour
Von Gruenigen (2012) [72] USA	*Aims*: 5% weight loss in 6 months. *Physical activity*: 150 min/week (five times/week for 30 min) for months 1 to 2, 225 min/week (five times/week for 45 min) for months 3 to 4 and 300 min/week (five times/week for 60 min) for months 5 to 6 and 10.000 steps per day or an increase of 2000 steps per day from baseline. *Diet*: improving diet quality by increasing fruits, vegetables, lean protein, whole grains and low-fat dairy intake and reducing saturated fat, simple carbohydrates and low nutrient/high calorie foods. *Who delivers the intervention*: A physician, a psychologist, a registered dietician and a physical therapist *How often*: 16 1-h group sessions (10 weekly followed by 6 bi-weekly). Three additional physician face-to-face counselling visits at 3, 6 and 12 months. Continued contact with dietician from 6 to 12 months via telephone, e-mail and newsletters. *For how long*: 6 months *In what format*: Group (8–10 women per group) and individual counselling; both face-to-face and via telephone, e-mail and newsletters. Participants were given pedometers, a physical activity guide, food/activity records and three-pound hand and adjustable ankle weights. *In what context*: not mentioned	The usual care group received an informational brochure (“Healthy Eating and Physical Activity Across Your Lifespan, Better Health and You”). Physician visits for the usual care group consisted of discussion of overall health concerns and review of medications and co-morbidities.	Social cognitive theory [73]	-Goal setting (behaviour) -Goal setting (outcome) -Action planning - Monitoring of outcome(s) of behaviour without feedback -Feedback on behaviour -Self-monitoring of behaviour -Biofeedback -Social support (unspecified) -Instruction on how to perform the behaviour -Credible source -Non-specific reward -Adding objects to the environment *Control group:* -Instruction on how to perform the behaviour

Randomized controlled trial with a usual care control, an attention control or a less intensive intervention control group; a feasibility or pilot study was excluded

^a^Intervention components included the following: ‘who delivered the intervention’, ‘how often’, ‘for how long’, ‘in what format’ and ‘in what context’ [26]. Intervention content was described by the behaviour change techniques that were used in the intervention. “To whom” the intervention was delivered is mentioned in Table 1

^b^Behaviour change techniques were coded according to the Behaviour Change Technique Taxonomy (v1) [28]

### Behaviour change technique coding

The Behaviour Change Technique Taxonomy version 1 (BCTTv1) was used to extract the BCTs that were used in the included interventions [28]. The BCTTv1 provides detailed definitions of 93 BCTs and includes examples of each BCT. This taxonomy has shown to be a reliable method for extracting information about intervention content and identifying potentially active ingredients associated with effectiveness [29, 30].

Two researchers (MH, MvS) independently coded intervention and control group content of all included interventions using the BCTTv1. Both coders were trained in applying the BCTTv1. When both coders independently coded the same BCT, the BCT was considered to be present. When only one of the coders coded a particular BCT, that BCT was discussed and only considered to be present if consensus was reached. When discrepancies could not be resolved through discussion, a third experienced coder was consulted (SM), and the BCT was considered to be present when two out of three coders deemed the BCT to be present.

When the authors of an included study referred to another publication for further details on intervention content, this other publication was also used to code BCTs. When authors of an included study mentioned that the content of their intervention was (partly) described elsewhere, but no reference was provided, only the description of the intervention content as mentioned in the included publication was used to code BCTs.

BCTs were identified and synthesized across interventions that were found to be effective after evaluation in an RCT with a usual care control, an attention control or a less intensive intervention control group. The frequency of identified BCTs was quantified across these effective interventions (see Table 3).

**Table 3:** Overview of behaviour change techniques and the frequency with which they have been used in included interventions that have shown to be effective after evaluation in a robust study

Behaviour change techniques^a^ (*N* = 30)	
Goal setting (behaviour)	8
Action planning	8
Social support (unspecified)	8
Instruction on how to perform the behaviour	8
Self-monitoring of behaviour	7
Adding objects to the environment	7
Goal setting (outcome)	6
Demonstration of the behaviour	5
Feedback on behaviour	5
Credible source	5
Behavioural practice/rehearsal	4
Graded tasks	4
Problem solving	4
Review behaviour goal(s)	3
Non-specific reward	3
Biofeedback	2
Self-monitoring of outcome(s) of behaviour	2
Reduce negative emotions	2
Discrepancy between current behaviour and goal	2
Review outcome goal(s)	1
Prompts/cues	1
Social reward	1
Information about health consequences	1
Monitoring of emotional consequences	1
Avoidance/reducing exposure to the behaviour	1
Framing/reframing	1
Self-talk	1
Framing/reframing	1
Monitoring of behaviour by others without feedback	1
Monitoring of outcome(s) of behaviour without feedback	1

Randomized controlled trial with a usual care control, an attention control, or a less intensive intervention control group; feasibility or pilot studies were excluded

^a^Behaviour change techniques were coded according to the Behaviour Change Technique Taxonomy (v1) [28]

## Results

**Fig. 1:**
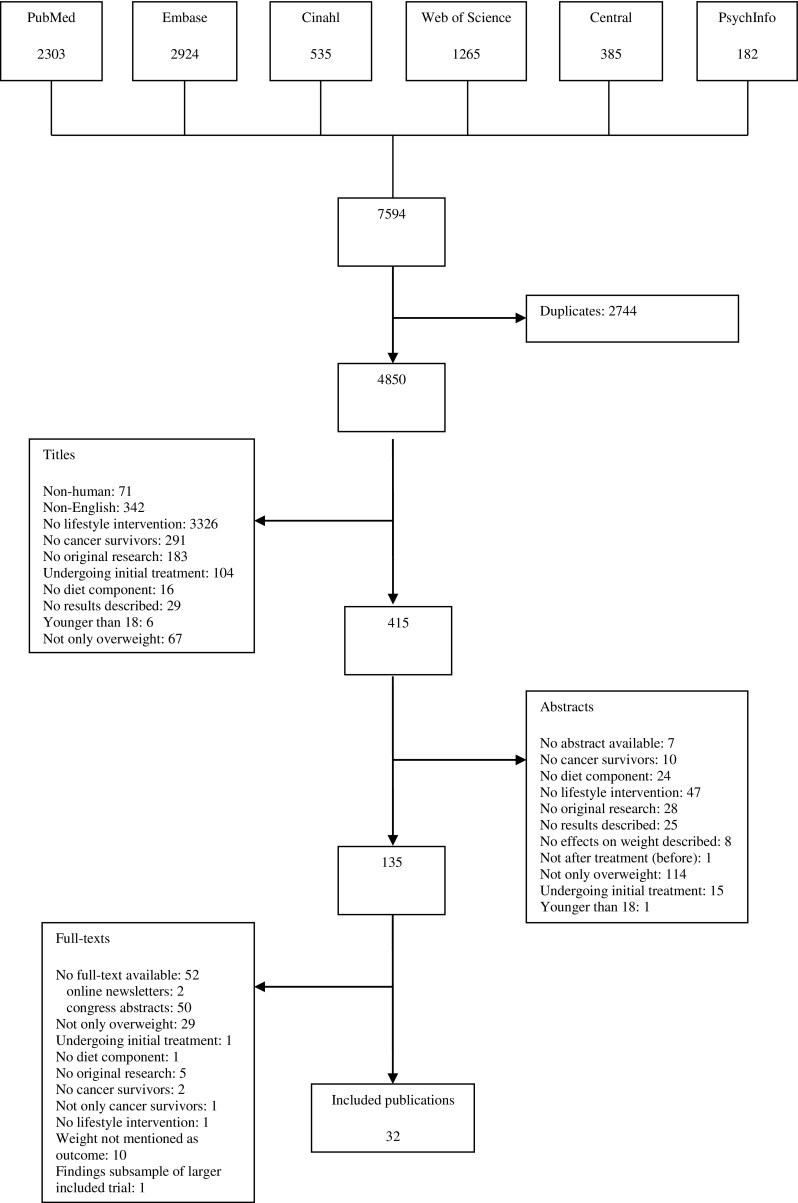
Flow chart of inclusion and exclusion of publications derived from the database searches

A flow diagram of the number of included and excluded papers is depicted in Fig. 1. In total, the database searches yielded 7594 references. After the removal of 2744 duplicates, 4850 titles and 415 abstracts were assessed for eligibility. Of the 135 full-texts that were screened, 103 references were excluded. See Fig. 1 for reasons for exclusion. Finally, 32 papers describing 27 interventions were included.

### General characteristics of the included intervention studies

Most of the included interventions were conducted in the USA (*n* = 22) [42–44, 46–59, 61–63, 65, 66, 69–72]. The other interventions were conducted in Canada [45], Spain [67], Italy [60], Australia [64] and the Netherlands [49] (see Table 1). The majority of the included interventions were offered to overweight breast cancer survivors (*n* = 17) [44–66, 68, 69, 79], of which five were offered to postmenopausal overweight breast cancer survivors [44–49]. The other interventions were offered to overweight endometrial cancer survivors (*n* = 2) [71, 72], overweight colorectal cancer survivors (*n* = 1) [41], a mixed study sample of overweight breast and endometrial cancer survivors (*n* = 1) [70] and a mixed study sample of overweight colorectal, breast and prostate cancer survivors (*n* = 1) [42, 43]. The number of participants varied from 10 [57] to 1510 [62] but was relatively low (*n* < 50) in the majority of the interventions (*n* = 16) [41, 44, 45, 48, 51–53, 55–57, 63–66, 68, 69, 71, 79]. Fifteen papers described 13 feasibility or pilot studies [41, 44, 45, 49–51, 55–57, 63, 64, 68–70, 79]. Intervention effects were assessed using an RCT (*n* = 16) [42, 43, 48–56, 58, 59, 61–63, 66, 71, 72], a single arm pretest-posttest design (*n* = 10) [41, 44, 45, 57, 60, 64, 65, 68–70, 79] or a three-arm non-RCT (*n* = 1) [46, 47]. Two of the 16 RCTs had a wait-list control design [42, 43, 53], two compared the effect of two different diets [48, 52], one assessed the additional effect of spirituality counselling in addition to a combined dietary and physical activity intervention [51], 10 had a usual care control, an attention control or a less intensive intervention control group (i.e. a robust study design) [50, 54–56, 58, 59, 61–63, 66, 71, 72] and in one RCT, no details about the control group was mentioned [49]. The three-arm non-RCT had a usual care control group [46, 47]. Effects were mostly assessed directly after the end of the intervention only (*n* = 19). However, in 8 out of the 27 intervention studies, a follow-up assessment after the end of the intervention was conducted, at 12 weeks [45], 6 months [53, 54, 64, 71, 72], 12 months [43] and 2 years after the end of the intervention [49].

### Effects on weight and weight loss maintenance

In 22 out of the 27 interventions, a statistically significant decrease in body weight was found directly after intervention completion [42–50, 53–61, 64–66, 68–72, 79], with a weight loss of <5% of baseline body weight after half of the effective interventions, a weight loss of 5–10% after 7 interventions [48, 54–56, 58–60, 64, 69] and a weight loss of ≥10% after 3 interventions [44, 46, 47, 68, 79]. Percent weight loss from baseline could not be calculated for one effective intervention, since mean baseline body weight was not reported [49]. Seven out of the eight intervention studies with a follow-up assessment after intervention completion showed a significant decrease in body weight at follow-up of 2.07 to 5.84% of baseline weight [43, 45, 49, 53, 64, 71, 72] (Table 1).

After exclusion of non-randomized studies and feasibility or pilot studies, nine robust studies describing eight interventions reported a statistically significant higher reduction in body weight in the intervention group compared with the control group directly after intervention completion [42, 43, 53, 54, 58, 59, 61, 66, 71, 72]. Contrary, one intervention was not found to be effective in inducing weight loss directly after intervention completion [62]. The amount of weight loss in the intervention group varied across these eight effective interventions from 2.4% [42] to 6.8% loss of baseline weight [58, 59]. A loss of <5% of baseline weight was found after intervention completion in six interventions [42, 53, 61, 66, 71, 72, 80], whereas weight loss of 5–10% was found after two interventions [54, 58, 59]. Two robust studies reported a significant higher reduction in body weight in the intervention group compared with the control group at 6-month follow-up after the end of two 6-month interventions [71, 72]. Both studies reported a mean decrease of ~3% of baseline body weight at follow-up in the intervention group [71, 72].

### Characteristics and intervention components of effective interventions

Characteristics and intervention components are described for the eight interventions that were effective in inducing weight loss after evaluation in a robust study [42, 43, 53, 54, 58, 59, 61, 66, 71, 72] (Table 2).

All interventions promoted both diet and physical activity to induce weight loss. Weight loss goals of these effective interventions varied across studies: five studies aimed for a specific weight loss goal (5–10%) [42, 43, 61, 75, 78], one study more generally aimed to facilitate a modest sustained weight loss without prescribing a percentage of weight loss [58, 59] and in three studies, no specific weight loss goal was mentioned [53, 54, 66].

A theoretical framework on which the intervention was based was reported in six out of eight interventions. Most interventions (*n* = 5) [42, 43, 54, 61, 71, 72] were based on ‘social cognitive theory’ [73]. Other theoretical frameworks included the ‘transtheoretical model’ [42, 43, 74], behavioural determinants model [61, 76], motivational interviewing [61, 77], cognitive behavioural therapy [58, 59, 61, 75, 78] and standard behavioural treatment for obesity [58, 59].

In most interventions, registered dieticians were involved in applying the intervention [54, 61, 66, 71, 72]. In one study, the intervention was applied by a dietician trained in exercise physiology and behaviour modification counselling alone [54]. In other studies, the dietician applied the intervention together with a ‘primary investigator’ [71], counsellors with backgrounds psychology and exercise physiology [61], exercise physiologists trained in medical rehabilitation [66] and with a ‘physician, a psychologist and a physical therapist’ [72]. Other interventions were applied by a ‘health counsellor’ [42, 43], an ‘instructor’ and a ‘trainer’ [53], as well as ‘trained investigators and research staff’ [58, 59].

Intervention duration varied from 12 weeks [66] to 24 months [61]. Duration of most interventions was 6 months (*n* = 4) [53, 54, 71, 72]. Frequency of intervention contacts decreased over time in the majority of these effective interventions [42, 43, 54, 58, 59, 61, 71, 72], mostly from weekly contacts in the first weeks or months of the intervention to monthly contacts towards the end of the intervention.

Five out of the eight interventions consisted of both individual and group counselling [53, 58, 59, 61, 71, 72], and three consisted of individual counselling only [42, 43, 54, 66]. Most interventions consisted of a combination of face-to-face and telephone counselling (*n* = 5) [53, 58, 59, 61, 71, 72]. Three of these interventions also included contacts via e-mail and/or newsletters [61, 71, 72]. One intervention consisted of contacts through telephone and mailed print materials only [42, 43], and in one study, it was determined by group assignment whether participants received face-to-face counselling only or telephone counselling only [54].

In three interventions, it was not mentioned where the intervention took place [58, 59, 71, 72]. One intervention was fully home-based [42, 43]. Three interventions where partly home-based [54, 61, 66] and also involved face-to-face meetings (e.g. at an exercise facility) [66]. One intervention was clinic based and included exercise sessions at a commercial fitness centre [53].

#### Content of effective interventions

Overall, 30 BCTs were used in the 8 effective interventions that were found to be effective after evaluation in a robust study (*n* = 8). The number of BCTs that were used in each effective intervention varied from 8 [54] to 18 [58, 59, 61], with a median number of 12.5 BCTs per intervention (Table 2). The BCTs ‘goal setting (behaviour)’, ‘action planning’, ‘social support (unspecified)’ and ‘instruction on how to perform the behaviour’ were used in all eight interventions. Other BCTs that were used in most of these effective interventions include the following: ‘self-monitoring of behaviour’ (*n* = 7), ‘adding objects to the environment’ (*n* = 7) and ‘goal setting (outcome)’ (*n* = 6) (Table 3).

## Discussion

This systematic review of the literature was the first to provide an overview of the effectiveness of lifestyle interventions for overweight survivors of any cancer type after completion of initial treatment. Although the majority of the included lifestyle interventions (22 out of 27) were found to be effective in reducing weight directly after intervention completion, relatively few of these interventions (*n* = 8) were evaluated in a robust study design. These robust studies generally showed a modest effect on weight (<5% loss of baseline body weight) and did not evaluate intervention effects on weight at long-term follow-up (≥12 months) after intervention completion. None of the interventions resulted in long-term weight loss maintenance. Our review was also the first to provide an overview of intervention components and characteristics of lifestyle interventions that have been found to be effective in reducing weight in overweight survivors of any cancer type after completion of initial treatment. Our review showed that all interventions that were found to be effective after evaluation in a robust study design promoted both diet and physical activity and used the BCTs goal setting (behaviour), action planning, social support (unspecified) and instruction on how to perform the behaviour. It also showed that effective interventions mostly combined group and individual counselling, had a duration of ≥6 months, combined face-to-face with non-face to face modalities (e.g. telephone counselling), were (co)applied by a registered dietician and were based on social cognitive theory.

Although our review is the first to report on the effects on weight of lifestyle interventions in overweight cancer survivors of any cancer type after completion of initial treatment, previous reviews have reported on the effects of lifestyle interventions in cancer survivors [32, 33, 35–38, 40, 81–83] and on effects on body weight in particular [35, 38, 81, 82]. However, these studies did not only include overweight cancer survivors and/or cancer survivors after completion of initial treatment and all included survivors of a specific cancer type only (breast cancer [35, 38, 82] and prostate cancer [81]). Additionally, other reviews on intervention effects among cancer survivors did not report effects on body weight [36, 37] or did not primarily focus on effects on body weight [32, 33, 40, 83].

Our finding that the vast majority of lifestyle interventions were effective in reducing weight in cancer survivors directly after intervention completion confirms the findings of previous reviews in breast cancer survivors [35, 38]. However, in these previous reviews, a larger proportion of effective interventions showed a weight loss of ≥5% of baseline weight directly after intervention completion (76.9% [38]; 61.5% [35]) compared with our review (25% of the 8 effective robust studies, and 47.6% out of all 22 effective interventions). The discrepancy between our finding on the proportion of effective interventions with a weight loss of ≥5% of baseline weight and the findings from these previous reviews in breast cancer survivors may be explained by the <5% weight loss observed directly after all effective interventions among survivors of other types of cancer than breast cancer (*n* = 5) and the <5% weight loss in all included interventions published after publication of the most recent review in 2014 [35] (*n* = 4). In addition, compared with reviews on intervention effects on weight among overweight or obese adults in the general population (showing a mean weight loss from baseline of 8.5–13%) [84–86], a lower percentage of weight loss from baseline was observed in our review (2.4–6.8%). The weight loss observed in our review is more in line with a recent review on the effects of weight loss interventions in overweight or obese adults with type II diabetes mellitus (17 of the 19 study groups reporting weight loss of <5%; mean of 3.2%) [87]. The authors suggested that it is generally more difficult for individuals with diabetes to lose weight and to maintain weight loss compared with individuals without diabetes [87]. Findings from our review may suggest that this might also be true for overweight cancer survivors. Disease- and treatment-related factors may hamper adherence to lifestyle recommendations in individuals diagnosed with an obesity-related disease, implying the need for a different behavioural strategy to reach sustained health behaviour changes.

As in our review, other reviews on the effects of lifestyle interventions in cancer survivors also found that few studies assessed weight at follow-up after intervention completion [35–37, 40]. Our findings on weight loss at follow-up after intervention completion are difficult to compare with these other reviews since effects on weight at follow-up after intervention completion were only briefly mentioned in these reviews, results were generally not expressed in percent weight loss from baseline, and either a different (less stringent) definition of long-term weight-loss maintenance was used or a definition of long-term weight-loss maintenance was not mentioned. In the literature on the effects of lifestyle interventions in overweight or obese adults, assessment of weight at long-term (≥1 year) follow-up after intervention completion is more common [86, 88]. Intervention studies in overweight and obese adults have generally shown that about half of initial weight loss is regained at ≥1 year follow-up after intervention completion [84–86, 88].

The findings from our review on characteristics and components used in effective weight loss interventions confirm previous research. Both Reeves et al. [35] and Playdon et al. [38] also reported that most interventions that led to clinically meaningful weight loss in breast cancer survivors combined counselling on diet, physical activity and behaviour modification. Furthermore, previous research in the general population has shown that combined diet and physical activity interventions are more often effective and provide a greater weight loss compared with a diet-only intervention [89–91]. Our finding that most effective interventions consisted of both group and individual counselling corresponds to previous findings that support both group and individual counselling for promoting weight loss for breast cancer survivors [38]. In line with our finding that most effective interventions used a combination of both face-to-face and non-face-to-face modalities including telephone counselling, Playdon et al. [38] reported that a greater proportion of interventions in breast cancer survivors resulting in ≥5% weight loss used both face-to-face counselling and telephone counselling. Our finding that the duration of most effective interventions was ≥6 months is in line with previous findings from Reeves et al. [35] suggesting that longer interventions (>6 months) achieved greater weight loss in breast cancer survivors. In addition, longer interventions have been associated with greater weight loss in obese or overweight adults [90].

Although the use of a theoretical framework has been reported to aid intervention development and evaluation and to promote insight into determinants of health behaviour change, no studies have directly tested different behavioural theories for weight loss in cancer survivors. Nevertheless, theory-based interventions are commonly used in cancer survivors. Findings from other reviews on the relation between the use of a theoretical framework and intervention effectiveness have been conflicting [29, 89, 92]. In line with our review, Stacey et al. [36] reported that SCT-based interventions appear effective in improving physical activity and a healthy diet in cancer survivors. In contrast, Playdon et al. [38] reported that few studies that resulted in >5% weight loss in breast cancer survivors based their intervention on a theoretical framework. Moreover, findings from Spark et al. [37] suggest that successful maintenance of physical activity and dietary outcomes in breast cancer survivors was more common in trials that were not based on a theoretical model. These conflicting findings may be due to an inadequate description of how theory is used in interventions. To promote a precise description of the theoretical base of interventions, a theory coding scheme can be used [93].

Although three other reviews on the effects of lifestyle interventions in cancer survivors have briefly reported on the BCTs used in these interventions [51, 56, 81], our review is the first to report on the BCTs used in each of the included interventions and to report on both the type and the number of BCTs that were used in effective interventions in cancer survivors. Moreover, our review is the first to report on the BCTs used in interventions that have been found to be effective in reducing weight in overweight survivors of different types of cancer after completion of treatment. These other reviews all used older versions of the BCT taxonomy [94, 95], used the BCT taxonomy with a different purpose [36], focused on physical activity and dietary outcomes rather than on weight [36, 37] and did not report on BCTs used in effective interventions [81]. Compared with our review, the only other review that reported on the number of BCTs used in effective interventions in cancer survivors found that less BCTs (median 5) were used in trials achieving successful maintenance of behaviour change outcomes [37]. Our findings with regard to the type of BCTs used in effective interventions confirm the finding of Stacey et al. [36] that the BCTs goal setting and self-monitoring of behaviour were commonly used in lifestyle interventions for cancer survivors. Findings from our review on the type of BCTs used in effective interventions confirm the results of previous research in the general overweight or obese population reporting that the BCTs social support, goal setting, self-monitoring of behaviour and ‘self-monitoring of outcomes of behaviour’ have been associated with intervention effectiveness [29, 89, 96]. Moreover, the BCTs self-monitoring of behaviour and self-monitoring of outcomes of behaviour have been associated with long-term weight loss maintenance [21, 97, 98].

### Methodological considerations

We comprehensively searched for relevant publications in six databases. However, we did not include non-English publications or unpublished literature, possibly resulting in a selection bias. During the database search, we excluded a considerable number of congress abstracts (*n* = 50). Half of these congress abstracts were not published as full-text papers at a later point in time, which may suggest a publication bias.

In line with recent reviews on the effects of lifestyle interventions in cancer survivors with mixed diagnosis [34, 40], the vast majority of the included interventions were offered to female cancer survivors. Therefore, our results may not be generalisable to male cancer survivors. Since our review aimed to provide a broad overview of the scientific literature on lifestyle interventions in overweight cancer survivors after completion of initial treatment, we did not only include high-quality studies. Of the included studies in our review, a relatively small proportion had a robust study design. Moreover, in line with other reviews on the effects of lifestyle interventions in cancer survivors [35, 36, 38, 81], sample sizes of the included studies were generally small. We did not conduct a quality assessment of the included studies. We did, however, focus on the studies with a robust study design in the interpretation of our findings.

Due to heterogeneity across included studies in timing, duration, intensity and content of the intervention, we did not conduct a meta-analysis to estimate a mean overall intervention effect on weight. Moreover, since only one out of the nine studies with a robust study design did not report a significant intervention effect, we could not compare components of effective and ineffective interventions. Although it is possible to detect patterns and generate testable hypotheses about likely effective components, there is not the power to be able to draw conclusions about effective intervention components. Therefore, our findings with regard to the intervention components used in effective interventions should be interpreted with caution. Finally, due to inadequate or incomplete description of intervention content in the included publications, the number and the variety of BCTs used in each intervention may have been underestimated.

#### Recommendations

To gain more insight into how long-term weight loss maintenance can be reached in overweight cancer survivors after completion of initial treatment, future robust studies should assess intervention effects at long-term follow-up (≥12 months) after intervention completion. A detailed description of the intervention should be part of systematic intervention development [99] and should be provided along with scientific publications regarding the intervention. This would be an important first step to be able to accumulate scientific evidence for effective intervention components and underlying behaviour change mechanisms. Furthermore, to promote comparability across intervention studies, we encourage researchers to use a BCT taxonomy [28] to describe the content of their intervention. Also, as part of systematic intervention evaluation [99], an extensive process evaluation should be conducted to gain more insight into effective intervention components and underlying behaviour change mechanisms [100]. These recommendations are particularly relevant for future studies, but may also, to some extent, be incorporated in current ongoing studies.

Although further research is needed on how to achieve long-term weight loss maintenance, oncologists and other healthcare professionals do not need to await these results and can refer to existing evidence-based lifestyle interventions to promote weight loss in overweight cancer survivors.

## Conclusions

Of the numerous studies that have shown that lifestyle interventions are effective in reducing weight in overweight cancer survivors after completion of initial treatment, the few studies with a robust study design generally showed a modest weight loss (<5% of baseline body weight) directly after intervention completion. There is a lack of knowledge on long-term effectiveness. All interventions that were found to be effective after evaluation in a robust study design promoted both diet and physical activity and used the BCTs goal setting (behaviour), action planning, social support (unspecified) and instruction on how to perform the behaviour. Our results on intervention components and characteristics of effective interventions could be used to inform intervention development or selection. To gain more insight into how long-term weight loss maintenance can be reached in overweight cancer survivors after completion of initial treatment, future publications should report on intervention effects on weight at ≥12 months after intervention completion and should include a detailed description of the intervention and an extensive process evaluation.

## References

[1] World Cancer Research Fund/American Institute for Cancer Research. Food, Nutrition, Physical Activity, and the Prevention of Cancer: a Global Perspective. Washington DC: AICR 2007.

[2] Azrad M, Demark-Wahnefried W (2014). The association between adiposity and breast cancer recurrence and survival: a review of the recent literature. Curr Nutr Rep.

[3] Calle EE, Rodriguez C, Walker-Thurmond K, Thun MJ (2003). Overweight, obesity, and mortality from cancer in a prospectively studied cohort of U.S. adults. N Engl J Med.

[4] Protani M, Coory M, Martin JH (2010). Effect of obesity on survival of women with breast cancer: systematic review and meta-analysis. Breast Cancer Res Treat.

[5] Ng AK, Travis LB (2008). Second primary cancers: an overview. Hematol Oncol Clin N Am.

[6] Riihimaki M, Thomsen H, Brandt A, Sundquist J, Hemminki K (2012). Death causes in breast cancer patients. Ann Oncol.

[7] Lipscombe LL, Chan WW, Yun L, Austin PC, Anderson GM, Rochon PA (2013). Incidence of diabetes among postmenopausal breast cancer survivors. Diabetologia.

[8] Lemasters T, Madhavan S, Sambamoorthi U, Kurian S. A population-based study comparing HRQoL among breast, prostate, and colorectal cancer survivors to propensity score matched controls, by cancer type, and gender. Psycho-oncology 2013. 10.1002/pon.3288 PMC4892175 23606210

[9] Weaver KE, Forsythe LP, Reeve BB, Alfano CM, Rodriguez JL, Sabatino SA (2013). Mental and physical health-related quality of life among U.S. cancer survivors: population estimates from the 2010 National Health Interview Survey. Cancer Epidemiology, Biomarkers & Prevention: a Publication of the American Association for Cancer Research, Cosponsored by the American Society of Preventive Oncology..

[10] Blanchard CM, Courneya KS, Stein K (2008). Cancer survivors’ adherence to lifestyle behavior recommendations and associations with health-related quality of life: results from the American Cancer Society’s SCS-II. J Clin Oncol: Off J Am Soc Clin Oncol.

[11] Kroenke CH, Fung TT, Hu FB, Holmes MD (2005). Dietary patterns and survival after breast cancer diagnosis. J Clin Oncol: Off J Am Soc Clin Oncol.

[12] Meyerhardt JA, Heseltine D, Niedzwiecki D, Hollis D, Saltz LB, Mayer RJ (2006). Impact of physical activity on cancer recurrence and survival in patients with stage III colon cancer: findings from CALGB 89803. J Clin Oncol: Off J Am Soc Clin Oncol.

[13] Meyerhardt JA, Niedzwiecki D, Hollis D, Saltz LB, Hu FB, Mayer RJ (2007). Association of dietary patterns with cancer recurrence and survival in patients with stage III colon cancer. JAMA: the Journal of the American Medical Association..

[14] Nichols HB, Trentham-Dietz A, Egan KM, Titus-Ernstoff L, Holmes MD, Bersch AJ (2009). Body mass index before and after breast cancer diagnosis: associations with all-cause, breast cancer, and cardiovascular disease mortality. Cancer Epidemiology, Biomarkers & Prevention: a Publication of the American Association for Cancer Research, Cosponsored by the American Society of Preventive Oncology..

[15] Kushi LH, Doyle C, McCullough M, Rock CL, Demark-Wahnefried W, Bandera EV (2012). American Cancer Society Guidelines on nutrition and physical activity for cancer prevention: reducing the risk of cancer with healthy food choices and physical activity. CA Cancer J Clin.

[16] Rock CL, Doyle C, Demark-Wahnefried W, Meyerhardt J, Courneya KS, Schwartz AL (2012). Nutrition and physical activity guidelines for cancer survivors. CA Cancer J Clin.

[17] World Cancer Research Fund/ American Institute for Cancer Research. Food, Nutrition, Physical Activity, and the Prevention of Cancer: a Global Perspective. Washington DC: AICR; 2007.

[18] Wing RR (2010). Long-term effects of a lifestyle intervention on weight and cardiovascular risk factors in individuals with type 2 diabetes mellitus: four-year results of the Look AHEAD trial. Arch Intern Med.

[19] Rossner S (1997). Defining success in obesity management. Int J Obes Relat Metab Disord.

[20] Wadden TA, Webb VL, Moran CH, Bailer BA (2012). Lifestyle modification for obesity: new developments in diet, physical activity, and behavior therapy. Circulation.

[21] Wing RR, Hill JO (2001). Successful weight loss maintenance. Annu Rev Nutr.

[22] Demark-Wahnefried W, Morey MC, Sloane R, Snyder DC, Miller PE, Hartman TJ (2012). Reach out to enhance wellness home-based diet-exercise intervention promotes reproducible and sustainable long-term improvements in health behaviors, body weight, and physical functioning in older, overweight/obese cancer survivors. J Clin Oncol.

[23] Mosher CE, Lipkus I, Sloane R, Snyder DC, Lobach DF, Demark-Wahnefried W (2012). Long-term outcomes of the FRESH START trial: exploring the role of self-efficacy in cancer survivors’ maintenance of dietary practices and physical activity. Psycho-Oncology.

[24] Robien K, Demark-Wahnefried W, Rock CL (2011). Evidence-based nutrition guidelines for cancer survivors: current guidelines, knowledge gaps, and future research directions. J Am Diet Assoc.

[25] Craig P, Dieppe P, Macintyre S, Michie S, Nazareth I, Petticrew M (2008). Developing and evaluating complex interventions: the new Medical Research Council guidance. BMJ.

[26] Davidson KW, Goldstein M, Kaplan RM, Kaufmann PG, Knatterud GL, Orleans CT (2003). Evidence-based behavioral medicine: what is it and how do we achieve it?. Ann Behav Med: Publ Soc Behav Med.

[27] Michie S, Fixsen D, Grimshaw JM, Eccles MP (2009). Specifying and reporting complex behaviour change interventions: the need for a scientific method. Implementation science: IS.

[28] Michie S, Richardson M, Johnston M, Abraham C, Francis J, Hardeman W (2013). The behavior change technique taxonomy (v1) of 93 hierarchically clustered techniques: building an international consensus for the reporting of behavior change interventions. Ann Behav Med: Publ Soc Behav Med.

[29] Michie S, Abraham C, Whittington C, McAteer J, Gupta S (2009). Effective techniques in healthy eating and physical activity interventions: a meta-regression. Health Psychol: Off J Div Health Psychol, Am Psychol Assoc.

[30] Presseau J, Ivers NM, Newham JJ, Knittle K, Danko KJ, Grimshaw JM (2015). Using a behaviour change techniques taxonomy to identify active ingredients within trials of implementation interventions for diabetes care. Implementation Science: IS.

[31] Abraham C, Wood CE, Johnston M, Francis J, Hardeman W, Richardson M (2015). Reliability of identification of behavior change techniques in intervention descriptions. Ann Behav Med: Publ Soc Behav Med.

[32] Pekmezi DW, Demark-Wahnefried W (2011). Updated evidence in support of diet and exercise interventions in cancer survivors. Acta Oncol.

[33] Demark-Wahnefried W, Jones LW (2008). Promoting a healthy lifestyle among cancer survivors. Hematol Oncol Clin N Am.

[34] Demark-Wahnefried W, Rogers LQ, Alfano CM, Thomson CA, Courneya KS, Meyerhardt JA (2015). Practical clinical interventions for diet, physical activity, and weight control in cancer survivors. CA Cancer J Clin.

[35] Reeves MM, Terranova CO, Eakin EG, Demark-Wahnefried W (2014). Weight loss intervention trials in women with breast cancer: a systematic review. Obes Rev: Off J Int Assoc Study Obes.

[36] Stacey FG, James EL, Chapman K, Courneya KS, Lubans DR (2015). A systematic review and meta-analysis of social cognitive theory-based physical activity and/or nutrition behavior change interventions for cancer survivors. Journal of Cancer Survivorship: Research and Practice..

[37] Spark LC, Reeves MM, Fjeldsoe BS, Eakin EG (2013). Physical activity and/or dietary interventions in breast cancer survivors: a systematic review of the maintenance of outcomes. J Cancer Survivorship: Res Pract.

[38] Playdon M, Thomas G, Sanft T, Harrigan M, Ligibel J, Irwin M (2013). Weight loss intervention for breast cancer survivors: a systematic review. Curr Breast Cancer Rep.

[39] Smits A, Lopes A, Das N, Bekkers R, Massuger L, Galaal K (2015). The effect of lifestyle interventions on the quality of life of gynaecological cancer survivors: a systematic review and meta-analysis. Gynecol Oncol.

[40] Goode AD, Lawler SP, Brakenridge CL, Reeves MM, Eakin EG (2015). Telephone, print, and web-based interventions for physical activity, diet, and weight control among cancer survivors: a systematic review. J Cancer Survivorship: Res Pract.

[41] Anderson AS, Caswell S, Wells M, Steele RJ, Macaskill S (2010). “It makes you feel so full of life” LiveWell, a feasibility study of a personalised lifestyle programme for colorectal cancer survivors. Support Care Cancer: Off J Multinatl Assoc Support Care Cancer.

[42] Morey MC, Snyder DC, Sloane R, Cohen HJ, Peterson B, Hartman TJ (2009). Effects of home-based diet and exercise on functional outcomes among older, overweight long-term cancer survivors: RENEW: a randomized controlled trial. JAMA: the Journal of the American Medical Association.

[43] Demark-Wahnefried W, Morey MC, Sloane R, Snyder DC, Miller PE, Hartman TJ (2012). Reach out to enhance wellness home-based diet-exercise intervention promotes reproducible and sustainable long-term improvements in health behaviors, body weight, and physical functioning in older, overweight/obese cancer survivors. J Clin Oncol: Off J Am Soc Clin Oncol.

[44] Befort CA, Klemp JR, Austin HL, Perri MG, Schmitz KH, Sullivan DK (2012). Outcomes of a weight loss intervention among rural breast cancer survivors. Breast Cancer Res Treat.

[45] Campbell KL, Van Patten CL, Neil SE, Kirkham AA, Gotay CC, Gelmon KA (2012). Feasibility of a lifestyle intervention on body weight and serum biomarkers in breast cancer survivors with overweight and obesity. J Acad Nutr Diet.

[46] Thompson HJ, Sedlacek SM, Playdon MC, Wolfe P, McGinley JN, Paul D (2015). Weight loss interventions for breast cancer survivors: impact of dietary pattern. PLoS One.

[47] Thompson HJ, Sedlacek SM, Wolfe P, Paul D, Lakoski SG, Playdon MC (2015). Impact of weight loss on plasma leptin and adiponectin in overweight-to-obese post menopausal breast cancer survivors. Nutrients.

[48] Thomson CA, Stopeck AT, Bea JW, Cussler E, Nardi E, Frey G (2010). Changes in body weight and metabolic indexes in overweight breast cancer survivors enrolled in a randomized trial of low-fat vs. reduced carbohydrate diets. Nutr Cancer.

[49] de Waard F, Ramlau R, Mulders Y, de Vries T, van Waveren S (1993). A feasibility study on weight reduction in obese postmenopausal breast cancer patients. Eur J Cancer Prev: Off J Eur Cancer Prev Organ (ECP).

[50] Demark-Wahnefried W, Jones LW, Snyder DC, Sloane RJ, Kimmick GG, Hughes DC (2014). Daughters and Mothers Against Breast Cancer (DAMES): main outcomes of a randomized controlled trial of weight loss in overweight mothers with breast cancer and their overweight daughters. Cancer.

[51] Djuric Z, Mirasolo J, Kimbrough L, Brown DR, Heilbrun LK, Canar L (2009). A pilot trial of spirituality counseling for weight loss maintenance in African American breast cancer survivors. J Natl Med Assoc.

[52] Flynn MM, Reinert SE (2010). Comparing an olive oil-enriched diet to a standard lower-fat diet for weight loss in breast cancer survivors: a pilot study. J Women’s Health (15409996).

[53] Greenlee HA, Crew KD, Mata JM, McKinley PS, Rundle AG, Zhang W (2013). A pilot randomized controlled trial of a commercial diet and exercise weight loss program in minority breast cancer survivors. Obesity (Silver Spring, Md).

[54] Harrigan M, Cartmel B, Loftfield E, Sanft T, Chagpar AB, Zhou Y et al.. Randomized trial comparing telephone versus in-person weight loss counseling on body composition and circulating biomarkers in women treated for breast cancer: the Lifestyle, Exercise, and Nutrition (LEAN) study. J Clin Oncol: Off J Am Soc Clin Oncol 2015. doi:10.1200/jco.2015.61.6375. 10.1200/JCO.2015.61.6375 PMC4872022 26598750

[55] Jen KL, Djuric Z, DiLaura NM, Buison A, Redd JN, Maranci V (2004). Improvement of metabolism among obese breast cancer survivors in differing weight loss regimens. Obes Res.

[56] Djuric Z, DiLaura NM, Jenkins I, Darga L, Jen CK, Mood D (2002). Combining weight-loss counseling with the weight watchers plan for obese breast cancer survivors. Obes Res.

[57] McTiernan A, Ulrich C, Kumai C, Bean D, Schwartz R, Mahloch J (1998). Anthropometric and hormone effects of an eight-week exercise-diet intervention in breast cancer patients: results of a pilot study. Cancer Epidemiology, Biomarkers & Prevention: a Publication of the American Association for Cancer Research, Cosponsored by the American Society of Preventive Oncology.

[58] Mefferd K, Nichols JF, Pakiz B, Rock CL (2007). A cognitive behavioral therapy intervention to promote weight loss improves body composition and blood lipid profiles among overweight breast cancer survivors. Breast Cancer Res Treat.

[59] Pakiz B, Flatt SW, Bardwell WA, Rock CL, Mills PJ (2011). Effects of a weight loss intervention on body mass, fitness, and inflammatory biomarkers in overweight or obese breast cancer survivors. Int J Behav Med.

[60] Patella MN, Ghiotto C, Pertile R, Fiduccia P, Bozza F, Pluchinotta A (2009). Effects of a nutritional intervention in overweight/obese breast cancer patients. Mediterr J Nutr Metab.

[61] Rock CL, Flatt S, Byers T, Colditz GA, Demark-Wahnefried W, Ganz PA et al.. Results of the exercise and nutrition to enhance recovery and good health for you (ENERGY) trial: a behavioral weight loss intervention in overweight or obese breast cancer survivors. Journal of Clinical Oncology.2015;33(15). 10.1200/JCO.2015.61.1095 PMC4582146 26282657

[62] Saquib N, Rock CL, Natarajan L, Flatt SW, Newman VA, Thomson CA (2009). Does a healthy diet help weight management among overweight and obese people?. Health Educ Behav: Off Publ Soc Public Health Educ.

[63] Sheppard VB, Hicks J, Makambi K, Hurtado-de-Mendoza A, Demark-Wahnefried W, Adams-Campbell L (2016). The feasibility and acceptability of a diet and exercise trial in overweight and obese black breast cancer survivors: the Stepping STONE study. Contemp Clin Trials.

[64] Spark LC, Fjeldsoe BS, Eakin EG, Reeves MM (2015). Efficacy of a text message-delivered extended contact intervention on maintenance of weight loss, physical activity, and dietary behavior change. JMIR mHealth and uHealth.

[65] Stolley MR, Sharp LK, Oh A, Schiffer L (2009). A weight loss intervention for African American breast cancer survivors, 2006. Prev Chronic Dis.

[66] Swisher AK, Abraham J, Bonner D, Gilleland D, Hobbs G, Kurian S (2015). Exercise and dietary advice intervention for survivors of triple-negative breast cancer: effects on body fat, physical function, quality of life, and adipokine profile. Support Care Cancer.

[67] Travier N, Fonseca-Nunes A, Javierre C, Guillamo E, Arribas L, Peiro I (2014). Effect of a diet and physical activity intervention on body weight and nutritional patterns in overweight and obese breast cancer survivors. Med Oncol (Northwood, London, England).

[68] Travier N, Guillamo E, Oviedo GR, Valls J, Buckland G, Fonseca-Nunes A (2015). Is quality of life related to cardiorespiratory fitness in overweight and obese breast cancer survivors?. Women & Health.

[69] Vitolins MZ, Milliron BJ, Hopkins JO, Fulmer A, Lawrence J, Melin S (2014). Weight loss intervention in survivors of ER/PR-negative breast cancer. Clin Med Insights Women’s Health.

[70] McCarroll ML, Armbruster S, Pohle-Krauza RJ, Lyzen AM, Min S, Nash DW (2015). Feasibility of a lifestyle intervention for overweight/obese endometrial and breast cancer survivors using an interactive mobile application. Gynecol Oncol.

[71] von Gruenigen VE, Courneya KS, Gibbons HE, Kavanagh MB, Waggoner SE, Lerner E (2008). Feasibility and effectiveness of a lifestyle intervention program in obese endometrial cancer patients: a randomized trial. Gynecol Oncol.

[72] von Gruenigen V, Frasure H, Kavanagh MB, Janata J, Waggoner S, Rose P (2012). Survivors of uterine cancer empowered by exercise and healthy diet (SUCCEED): a randomized controlled trial. Gynecol Oncol.

[73] Bandura A (1986). Social foundations of thought and action: a social cognitive theory.

[74] Prochaska JO, Velicer WF (1997). The transtheoretical model of health behavior change. Am J Health Promot: AJHP.

[75] Cooper Z, Fairburn CG. Cognitive-behavioral treatment of obesity: a clinician’s guide: Guilford Press; 2003.

[76] Sallis JF, Calfas KJ, Alcaraz JE, Gehrman C, Johnson MF (1999). Potential mediators of change in a physical activity promotion course for university students: Project GRAD. Ann Behav Med: a Publ Soc Behav Med.

[77] Miller WR, Rollnick S (1991). Motivational interviewing: preparing people to change addictive behavior.

[78] Cooper A, Fairburn CG, Hawker DM (2003). Cognitive-behavioral treatment of obesity.

[79] Travier N, Fonseca-Nunes A, Javierre C, Guillamo E, Arribas L, Peiro I (2014). Effect of a diet and physical activity intervention on body weight and nutritional patterns in overweight and obese breast cancer survivors. Med Oncol.

[80] Haggerty AF, Allison K, Sarwer DB, Spitzer J, Raggio G, Chu C (2014). The use of technology-based weight loss intervention for endometrial cancer survivors with obesity. Gynecol Oncol.

[81] Mohamad H, McNeill G, Haseen F, N'Dow J, Craig LC, Heys SD (2015). The effect of dietary and exercise interventions on body weight in prostate cancer patients: a systematic review. Nutr Cancer.

[82] Rooney M, Wald A (2007). Interventions for the management of weight and body composition changes in women with breast cancer. Clin J Oncol Nurs.

[83] Spencer JC, Wheeler SB (2016). A systematic review of motivational interviewing interventions in cancer patients and survivors. Patient Educ Couns.

[84] Kouvelioti R, Vagenas G, Langley-Evans S (2014). Effects of exercise and diet on weight loss maintenance in overweight and obese adults: a systematic review. J Sports Med Phys Fitness.

[85] Franz MJ, VanWormer JJ, Crain AL, Boucher JL, Histon T, Caplan W (2007). Weight-loss outcomes: a systematic review and meta-analysis of weight-loss clinical trials with a minimum 1-year follow-up. J Am Diet Assoc.

[86] Barte JC, ter Bogt NC, Bogers RP, Teixeira PJ, Blissmer B, Mori TA (2010). Maintenance of weight loss after lifestyle interventions for overweight and obesity, a systematic review. Obes Rev: Off J Int Assoc Study Obes.

[87] Franz MJ, Boucher JL, Rutten-Ramos S, VanWormer JJ (2015). Lifestyle weight-loss intervention outcomes in overweight and obese adults with type 2 diabetes: a systematic review and meta-analysis of randomized clinical trials. J Acad Nutr Diet.

[88] Curioni CC, Lourenco PM (2005). Long-term weight loss after diet and exercise: a systematic review. Int J Obes.

[89] Greaves CJ, Sheppard KE, Abraham C, Hardeman W, Roden M, Evans PH (2011). Systematic review of reviews of intervention components associated with increased effectiveness in dietary and physical activity interventions. BMC Public Health.

[90] Wu T, Gao X, Chen M, van Dam RM (2009). Long-term effectiveness of diet-plus-exercise interventions vs. diet-only interventions for weight loss: a meta-analysis. Obes Rev: Off J Int Assoc Study Obes.

[91] Kirk SF, Penney TL, McHugh TL, Sharma AM (2012). Effective weight management practice: a review of the lifestyle intervention evidence. Int J Obes.

[92] Prestwich A, Sniehotta FF, Whittington C, Dombrowski SU, Rogers L, Michie S (2014). Does theory influence the effectiveness of health behavior interventions? Meta-analysis. Health Psychol: Off J Div Health Psychol, Am Psychol Assoc.

[93] Michie S, Prestwich A (2010). Are interventions theory-based? Development of a theory coding scheme. Health Psychol: Off J Div Health Psychol, Am Psychol Assoc.

[94] Abraham C, Michie S (2008). A taxonomy of behavior change techniques used in interventions. Health Psychology: Official Journal of the Division of Health Psychology, American Psychological Association.

[95] Michie S, Ashford S, Sniehotta FF, Dombrowski SU, Bishop A, French DP (2011). A refined taxonomy of behaviour change techniques to help people change their physical activity and healthy eating behaviours: the CALO-RE taxonomy. Psychol Health.

[96] Lara J, Evans EH, O'Brien N, Moynihan PJ, Meyer TD, Adamson AJ (2014). Association of behaviour change techniques with effectiveness of dietary interventions among adults of retirement age: a systematic review and meta-analysis of randomised controlled trials. BMC Medicine.

[97] Soleymani T, Daniel S, Garvey WT (2016). Weight maintenance: challenges, tools and strategies for primary care physicians. Obes Rev: Off J Int Assoc Study Obes.

[98] Montesi L, El Ghoch M, Brodosi L, Calugi S, Marchesini G, Dalle GR (2016). Long-term weight loss maintenance for obesity: a multidisciplinary approach. Diabetes, Metabolic Syndrome and Obesity: Targets and Therapy.

[99] Bartholomew LK, Parcel GS, Kok G (1998). Intervention mapping: a process for developing theory- and evidence-based health education programs. Health Education & Behavior: the Official Publication of the Society for Public Health Education.

[100] Linnan L, Steckler A (2002). Process Evaluation for Public Health Interventions and Research.

